# Lessons learned from lockdown: how the COVID-19 pandemic revealed intersecting inequalities of mental health, well-being, and learning for first-year UK university students

**DOI:** 10.1080/17482631.2026.2624011

**Published:** 2026-02-03

**Authors:** Charlotte Horner, Siobhan Hugh-Jones, Cathy Brennan, Ed Sutherland

**Affiliations:** a Leeds Institute of Health Science, University of Leeds, Leeds, UK; b Psychology, University of Leeds, Leeds, UK

**Keywords:** Mental health, isolation, pandemic, university students, inequality, longitudinal

## Abstract

**Purpose:**

Many COVID-19 studies treat the student population as homogenous, concealing the experiences of vulnerable groups. This study conceptualised vulnerability during the pandemic as an intersection of being a first-year student with a history of poor mental health and being from a low-income background. The aim of this study was to understand how these students' profiles shape their university and educational experience over 1 year of the pandemic.

**Methods:**

Longitudinal, semi-structured interviews with 20 first-year students from UK universities were conducted during the 2020–2021 academic year. The interview data were analysed using Interpretative Phenomenological Analysis (IPA).

**Results:**

Themes were (i) *(Not) managing mental health impacts*, where participants expressed a sense of barely surviving; (ii) *little choice, more risk, and more isolation*, where low-income students reported struggling to balance the risk of illness with employment; and (iii) *Past mental health experiences: Feeling more isolated and forgotten,* where previous experiences of poor mental health left students vulnerable to a spiralling state of poor mental well-being.

**Discussion:**

This study identified how vulnerabilities intersect and interact with challenging circumstances to reveal how those inequalities were experienced by students. Recommendations were made to support students by improving visibility and access to mental health services.

## Introduction

In its first-ever Human Development Report devoted entirely to education, the World Bank announced a global learning crisis during the COVID-19 pandemic, stating that the public was set to “face a generational catastrophe that could waste untold human potential, undermine decades of progress, and exacerbate entrenched inequalities” (UNESCO, 2020, p. 6). This concern has been subsequently echoed in a March 2025 UNESCO report of the 5-year impact of COVID-19, where it is highlighted in the Global Education Coalition report that educational institutes must continue to “prioritize crisis response and resilience building” as the long-term impacts of the pandemic continue to be felt (UNESCO, 2025). The report attributes these amplified inequalities to a “hobbling” of disadvantaged young people who were not given the educational boost they needed to succeed by the learning institutions they depended upon (UNESCO, 2025).

Mental health conditions among students are associated with inequalities (e.g. unequal access to resources) and unequal outcomes (e.g. not graduating, lower employment rates) (Dougall et al., 2023; Robertson et al., 2022), and these inequalities are often heightened in those with intersectional identities (Ali & Khattak, 2023). Rates of mental health conditions are disproportionately represented in groups with intersectional identities, often connecting to gender, race/ethnicity, disability, and socioeconomic status (SES) (Salimi et al., 2023).

Those from disadvantaged backgrounds are especially affected by overlapping points of disadvantage. A person's SES has well-documented impacts on mental health outcomes as those from less-affluent areas cannot access care as easily as those in affluent areas (Coombs et al., 2021) and are more vulnerable to poor mental health overall (Claes et al., 2021). Even with access to means-tested financial support, disadvantage has been associated with anxiety and stress in first-year university students (McCloud, 2022; Richardson et al., 2024). As this literature suggests, being a first-year student in financial hardship and/or with prior or existing mental health difficulties constituted a triple whammy of vulnerability during this time. The pandemic served to exacerbate pre-existing inequalities and impacted certain groups disproportionately. It has also opened new inequality gaps along dimensions that were previously less significant, such as socializing and working from home. All of these stressors could equate to a strain on each student's mental health greater than that of their less-vulnerable peers.

Universities hold power and could play a vital role in preventing and mitigating inequalities emerging from, for example, SES, disability, or race/ethnicity (Farquharson et al., 2024). However, it is argued that universities are not doing enough is being done to understand and redress such inequalities (Coyne et al., 2020). This is particularly pertinent to the UK university environment as they have a duty, enshrined within the Equality Act (2010), to make reasonable adjustments for those who may experience disadvantage and/or inequality (Government Equalities Office, 2015). Without appropriate knowledge of these inequalities, it could be argued that needed adjustments could not be made.

We use the term equality as opposed to equity to reflect a focus on the expected uniformity of access to higher education resources and opportunities during the COVID-19 pandemic. While equity emphasizes fairness through differential support tailored to individual needs (Braveman & Gruskin, 2003), equality refers to the provision of the same levels of support and opportunity for all individuals regardless of background (Espinoza, 2007). Given the study's emphasis on students' perceptions of access and participation during the pandemic, the concept of equality is more appropriate for assessing different experiences of the same educational provision. This allows for a critique of whether the UK higher educational system provided the same opportunities to all students during the unprecedented context of the pandemic. Nonetheless, equity remains important to consider, as many of the challenges faced by disadvantaged students were inherently rooted in pre-existing structural inequalities that require purposeful action to resolve (Gillborn et al., 2012). Thus, while equality frames the central analytic lens of this study, it is understood that equity-oriented solutions are essential for addressing the deeper systemic issues exposed by the pandemic.

Such literature demonstrates a substantial need to explore the unprecedented and unique state of stress for university students during the pandemic, including abrupt transitions to remote learning, prolonged social isolation, and heightened academic and financial uncertainty. Although emerging research has identified elevated levels of anxiety, depression, and stress among students during this period (Huckins et al., 2020; Son et al., 2020), current evidence can often be inattentive to contextual and institutional factors that shape mental health outcomes and could be better studied with qualitative methods (Roberts et al., 2021). By addressing these gaps, the present study will contribute to the literature by offering a more nuanced, empirically grounded understanding of how pandemic-related disruptions influence student well-being, thereby informing theory, guiding targeted interventions, and strengthening institutional preparedness for future crises.

The aim of this study, therefore, building on the author's original thesis (Horner, 2023), was to explore how students with vulnerable profiles were impacted by the pandemic, and what lessons could be learnt to support the development of effective structures that could work to reduce inequalities in outcomes in post-pandemic in universities in the UK.

## Method

### Ethical considerations

Ethical approval was gained from the University of Leeds, Faculty of Medicine & Health Research Ethics Committee [PSYC-147, 23 November 2020].

### Study design

This study used a longitudinal, exploratory design involving three qualitative semi-structured interviews with the same university students conducted over 1 year and analyzed via interpretative phenomenological analysis (IPA), a systematic, idiographic approach to the analysis of rich, lived experience data, including the meaning that participants give to those experiences (Smith et al., 2021). This responded to the call from O'Connor et al. (2020) for qualitative, longitudinal research to detail the long-term psychological impacts of the pandemic. The use of semi-structured interviews allowed the researcher flexibility to respond to each participant individually and more freely explore their lived experiences (Subu and Wati, 2021)—in turn encouraging the generation of rich data for the study's purposes. It also allowed for an iterative process where interview questions could be modified throughout the study in response to emerging topics and themes important to participants.

IPA differentiates itself from other qualitative analysis methods through its idiographic focus on individual meaning-making within the study context (Eatough & Smith, 2017). IPA does not seek to establish a generalizable truth, which melds well with the longitudinal qualitative research orientation in that they both assume an ontological understanding of lived experiences as subjective and ever-changing.

Those with lived experience were involved with both the design and analysis of this study. The lead researcher and a member of the analysis team were members of the target population in the past, namely being a university student from a disadvantaged background, with periods of reduced mental well-being. The lead researcher shaped the study from design to completion by writing interview guides, conducting interviews, coding transcripts and identifying themes; the analysis team members focused on coding and theme generation. Coming from a position of empathy and understanding allowed the study design to be conscious of specific challenges of this population and helped to assure participant autonomy and dignity throughout the process.

Rigour in this study was addressed through the triangulation of multiple researcher perspectives during the data interpretation process to enhance credibility, dependability, and confirmability. While double hermeneutic is explicit in IPA (Smith et al., 2014), incorporating multiple analysts helped to challenge individual assumptions and reduce the potential for singular bias. Interpretative triangulation, in particular, involved collaborative coding sessions and discussions among the research team to help ensure consistency and consider alternative perspectives (Richards & Hemphill, 2018). This process did not seek consensus for its own sake but aimed to enrich the analysis through the integration of different viewpoints and disciplinary lenses, as members of the analysis team had backgrounds in both research and clinical settings.

Additionally, reflexive field notes were used throughout the interview process for the lead researcher to record and reflect on any particular behaviour or conversation points that appeared to be significant (Phillippi & Lauderdale, 2018). While these were not analyzed, they were used to aid in the construction of rich data supported by contextual information, and to prompt the researcher to closely observe the behaviour and environment of each participant.

### Participants and study setting

Participants were sought via social media due to lockdowns. Inclusion criteria were first-year students at a UK university in receipt of a full student maintenance loan (as a proxy for low SES) and self-reporting a period of poor mental health of between 3 and 12 weeks in the past 6 months. Participants would also be asked to be available for the full duration of the study, with interviews taking place at Time Point 1 (T1, December 2020), Time Point 2 (T2, March 2021), and Time Point 3 (T3, June 2021). Students with a mental health diagnosis or receiving ongoing professional support were excluded for safety reasons. All participants provided signed informed consent prior to the interviews and received renumeration for their time.

At T1, we met our target sample size of 20, which is suitable for a longitudinal qualitative study and anticipates some attrition (Gustavson et al., 2012). In terms of diversity, the sample was comprised of majority (55%) White/British female participants, which is the most common demographic of UK university students according to data from Higher Education Statistics Agency; in this, the present study was representative of the university population. Additionally, 20% (*n* = 4) of participants entering the study at T1 were Asian, which further reflects on the UK university population during the 2020 academic year where 12.2% of new undergraduate students were Asian.

It was acknowledged that no Black/Black British students took part; however, given the long-term commitment the study required and the focus on poor mental health, the aim of seeking participants was always intended to draw as many potential participants as possible as opposed to imposing targets of ethnicity, which could have resulted in turning otherwise-eligible students away who wanted to share their experiences. In terms of sexuality, 30% (*n* = 6) of participants identified as either homosexual, bisexual, or other which offered a more diverse participant pool when compared to the number of UK university students who self-identify as non-heterosexual; 3.3–7.5% according to 2020 intake statistics (UCAS, 2021).

Table I shows participant details and interviews completed. Although we stated that participants with a mental health diagnosis were not eligible, during the interview participants reported symptoms of depression (*n* = 19), anxiety (*n* = 17), eating disorders (*n* = 1), anger (*n* = 2), and attention regulation difficulties (*n* = 2).

**Table I.** Participant demographics by interview time point (T).

Characteristic		T1	T2	T3
Gender	Male	5	4	2
	Female	15	14	14
	Non-binary	0	0	0
Sexual identity	Heterosexual	14	12	11
	Homosexual	2	2	1
	Bisexual	3	3	3
	Other	1	1	1
Ethnicity	White/White British	14	13	11
	Asian/Asian British	4	3	3
	Black/Black British	0	0	0
	Other	2	2	2
University location	Leeds	12	11	10
	London	2	2	2
	Wolverhampton	1	0	0
	Salford	1	1	0
	Bristol	3	3	3
	Lincoln	1	1	1
Number of participants		20	17	15

### Data collection

**Figure 1.**
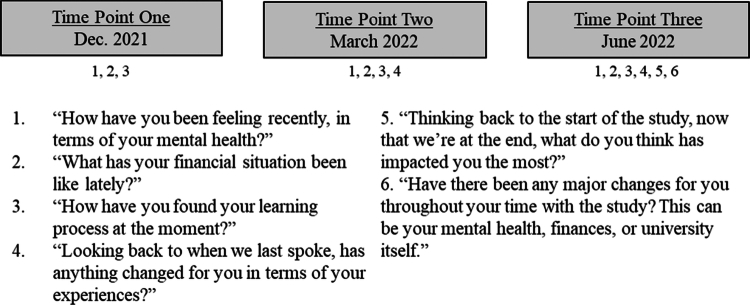
Key interview questions mapped at each time point.

In order to generate rich data on lived experience, and in anticipation of using IPA we created a topic guide which could be used flexibly to allow issues raised by interviewee: (i) how their mental health was impacted by the pandemic; (ii) how the pandemic impacted their financial situation; (iii) how they managed remote and online education; and (iv) how they had coped during the pandemic. Individual cases were examined individually and systemically to identify key changes in their experiences and explore how those changes related to their transcripts. The interviews were conducted by the first author, who is experienced in interviewing students about mental health; the first author was trained in interview methods at the undergraduate and postgraduate levels and completed both as lead interviewers and analysts. Ethical practice and safeguarding training were also completed as part of professional development prior to this study. See Figure 1 for a list of central interview questions used to explore participants' lived experiences across time points.

Interviews were conducted online via MS Teams and participants could choose to be interviewed via live chat (camera on/off) or via the live chat box. Such methods have been scrutinized for effectiveness during the COVID-19 era of remote research and were found to facilitate rich data for analysis (Engward et al., 2022). A total of 52 interviews were conducted across the three time points, with an average duration of 50–90 min. The interviews were recorded and transcribed by the lead researcher and saved securely on a password-protected one drive folder.

### Data analysis

The driving analytic question was “What was the lived experience of participants during the pandemic, especially in terms of their learning, mental health and financial circumstances?” We followed five steps of IPA from Miller et al. (2018). Analysis was led by the first author and supported by an analysis team (as per Guest & MacQueen, 2008). IPA began with data familiarization then open coding to capture either semantic (e.g. “difficulty budgeting”) or latent (e.g. “shadow of anxiety”) meaning (Finlay, 2011). Each interview was analyzed separately by a primary then a secondary coder who interrogated the provisional theme and worked with the primary coder to deepen or refine the themes. Connections between themes were explored across the dataset to develop a final set of themes (Smith et al., 2021).

## Results

In total, 15 participants completed all interviews across three time points. The sample were mostly (55%) White British, female students, with 20% being Asian responders. In terms of sexuality, 30% identified as bisexual, homosexual, or other. Three main themes were generated that explored the intersectionality and inequalities of mental health, economic status, and learning. These are outlined below with interview quotes that are identified by gender and time point (for example, F, T2).

(Not) Managing mental health impacts
Little choice, more risk, more isolation
Past mental health experiences: Feeling more isolated and forgotten

### Theme 1: (not) managing mental health impacts

Worsening mental health during their first year of university was reported by most participants, often via the metaphor of “sliding back”. This suggested that their pre-pandemic climb to recovery had been voided or reduced as previous difficulties resurfaced. For example, this male participant explained how lockdowns prevented him accessing his usual coping mechanisms and his prior experiences with depressive and anxious thoughts returned. He felt as though he had let himself down by thinking such things again:

“not being able to live at all right now [means] I slide back into all of the issues I used to have. […] The biggest things that helped me get better and stay better after I had been ill before I haven't been able to do. I am back into all the negative ways I was thinking before […] it's destroyed any motivation I've had.” (M, T1)

Similarly, the female below described how leaving home to go to university, with newfound independence and freedom, was positive for her mental health. Having to return home due to lockdowns was the vulnerability:

“moving back home puts me back to square one […] I've lost my independence […] my room doesn't feel like my room anymore and my head doesn't feel like mine because it's like I'm still in my childhood bedroom. I have so much more work to do now since the proper year has started. Everything's come at once.” (F, T2)

She described how alien her home, and then her mind, felt back in a childhood space. Her account signalled disorientation, weirdness of experience and of real setbacks in her mental health based on place (home) and its memories and lack of autonomy. Notable here is that she felt strongly that the deterioration in her mental health was driven purely by where she was physically and not by her own mental state per se, showing how location and space matter. She also highlights that the changing demands of work between T1 and T2 have made these feelings even worse, as she has a great academic load to manage. Others talked about the impact of being prevented from managing their anxiety through positive social challenges. Lockdown gave them a reason to not leave their home, meaning that their avoidance behaviours went unchallenged and that their ability to cope with social life was sliding back. While this provided short-term relief, they knew that lockdowns would end, adding to anxiety, as this participant explained:

“to be honest I think I always looked for reasons to not leave the house so Covid 19 was a good excuse. [… ] It's like the months I've spent not having to go into university have made knowing that I'll have to eventually even worse. I'm so much more anxious […] I don't know if it would have been easier or not […] So much second guessing and supposing that it makes it hard to move on the way I used to.” (F, T2)

Here is conveyed the mental gymnastics that came with living with anxiety during the pandemic, and a confusion about whether “better” for her would have been the social challenge or the respite from it. Significantly, she was concerned about her ability to *“move on the way I used to”,* indicating unrecoverable past progress in managing her mental health.

### Theme 2: little choice, more risk, more isolation

This theme represents the two main ways in which students perceived inequalities in relation to students who were not on a low income during the pandemic, and how being on a low income meant missing out on typical student experiences. Most participants could not rely on parents or guardians for financial assistance and had part-time jobs during the pandemic. They felt they had little choice but to put their health at risk by going to work and being exposed to infection, and some discussed comparing themselves negatively to their peers—believing themselves to be lesser as a result of financial burdens:

“I've always worried about money […] even with Covid I don't really have a choice because if I don't work. I don't get to really eat or do anything. It feels like I'm just less than everyone else since they do so much more than me.” (F, T2)

For these students, work was not for “pocket money” but for subsistence money. The impact of the pressures to work in risky pandemic environments was compounded by their mental health; participants felt anxious about going out, but also about staying in. Some students who were living at home during lockdowns also worried about the risk they were exposing their family to, and how they had to weigh this against the reality of needing a living wage, as this participant explained:

“If I want more hours they would have to be in person. So there is this balance between putting myself and my family at risk of getting Covid… It definitely makes me more anxious as I have to weigh my decisions to go out against my safety to do so, etc. My anxiety is definitely linked to wanting to keep family safe, but also wanting to support myself.” (F, T1)

These feelings of financial anxiety were further compounded by the perceived unfairness that they were paying the usual fees, but could only access online material, and were only being taught remotely. Given that these students had pre-existing experiences of poor mental health, feeling down or prone to more critical/negative thoughts, the strong emotions of disappointment, frustration, worry, etc. were difficult to process. These students felt that they were getting into a huge amount of debt, with ultimately little student experience to speak of.

“I said to the lecturer, […] can you please direct me to some online resources that can help with this? [She said] YouTube it. I just found it so insulting […] I get angry just thinking about it because here I've come to you, said I'm struggling. I need support and all you're saying is go find that support yourself elsewhere […] I'm not paying £ 9000 a year for that. Like I'm getting into debt for this.” (F, T2)

Given that these students were already experiencing a sense of otherness due to their financial circumstances, the additional stressor of potentially unsympathetic authority figures added to the strain on their mental health. This, in turn, meant that these students had fewer cognitive resources available to devote to their studies when so much energy was diverted to simply managing their day-to-day finances and mental health:

“I just don't have it in me. It's like I work 9 or 11 hour shifts sometimes and then I'm totally drained when I get home. Do they want me to do [university] work until 5 in the morning and then go to lectures at 8 ? It's crazy, like my brain is just done, out, and I can't feel anything else […] I can't handle it. I literally can't afford to be okay.” (M, T2)

This participant shared an experience similar to several others, where the combined demands of employment, university work and their own mental health were too much to manage all at once. These students had no choice but to push through, regardless of the impact on their well-being. Unable to afford the financial cost of prioritizing their mental health, these experiences highlight the differing experiences of those who had to work, and those who did not.

Participants were also forced to commit to a complete readjustment and restart to themselves and their routine as lockdown constantly changed which, while being challenging for all students, impacted participants particularly owing to their past struggles with mental health. Such experiences highlight how financial strain intersects with well-being as social connections, so critical for mental health, had an associated financial cost that some could not afford. This brought not only a sense of “missing out” but also “falling behind” in forging new friendships, another critical dimension of university life accessed by those with resources.

### Theme 3: past mental health experiences: feeling more isolated and forgotten

Past mental health stressors made these students more vulnerable to feeling isolated and forgotten by both their institution—whom they relied on to provide essential support—and their peers—who seemed to be coping much better with the challenges they were faced with. In particular, students found it difficult to cope with the lack of social energy and support that they would otherwise access:

“I'm a very sociable person so it's very essential for me to stay in touch with people […] And this year I've been almost completely deprived of that. I just don't have any real friends and I feel like I can't make any.” (F, T1)

This experience highlights how significant an issue this lack of social interaction was, going on to say that she felt “too anxious” to reach out without additional support. That she feels “deprived” highlights the sense of loss of these potential social interactions, and her sense of isolation and loneliness at being unable to interact with other people.

Isolation in particular was distressing for all participants as they felt caught in a cycle of sadness and loneliness. Many felt that the world around them, including their university institute and peers, had forgotten them, and this led to losing confidence in themselves and in their ability to learn and succeed at university:

“I've felt lonely as I haven't been able to see or make friends. I struggle with social situations anyway, and this has made me feel worse. It feels like everything is on hold and I'm not making progress because the outcome of my time studying and engaging with university resources online is intangible… I feel like I've been forgotten.” (F, T2)

This participant encapsulates the overarching qualities of this theme in her experience where she describes the loneliness that being forgotten—from her perspective—has caused. The sense that she is trapped and “on hold”, unable to progress because of things beyond her control, resonated with many other participants who shared similar views. She also highlighted the “intangible” nature of online learning, where the added distance between herself, her peers and lecturers only heighten these feelings of isolation, loneliness, and the sense of being forgotten. Moreover, her anxiety increased as a result of the long period of isolation, leaving her less likely to reach out in future.

These experiences had some potentially serious consequences for the more vulnerable members of this study. Individuals with prior experience of particularly high levels of depression were more susceptible to these feelings of abandonment—especially when promises of individual support were not followed through:

“At first, I had been given promises of visitation and a sense of normality from both the university and the people in my life, but each and every announcement meant that they couldn't live up to the promises that were made to me.” (F, T3) going on to say that had she not fought to help herself, she “would've been pushed to act the way I had [before] - which resulted in self-harm”.

That a student came close to repeating self-harm highlights how impactful and strong emotions were, especially when coupled with an already vulnerable mental state. In this case, it was the sense of being let down, rather than isolation per se, that was so difficult to manage—though the two experiences interacted by making each other worse.

## Discussion

This study investigated the lived experience of vulnerable university students undergoing a key transition during the COVID-19 pandemic and UK lockdown, with particular focus on how the pandemic affected students experiencing overlapping vulnerabilities: past experience of poor mental health, being a low-income student, and being at a major life transition point in becoming a first year university student. It was hoped that new insights to inequality would be made through using the pandemic as a magnifying glass to highlight such issues and suggest ways that these inequalities could be mitigated.

### Mental health intersections

Participants spoke about how their mental health had been negatively impacted during the pandemic, and how they felt that they were only just treading water in terms of their overall well-being and academic achievement. The most common and resounding factor throughout much of the participants' experiences was that of isolation, and its profound impact on their mental health. This experience echoes other cross-sectional work that explored psychological stressors of the pandemic, many of which identified isolation as one of the more significant factors impacting university students during that time (Leal Filho et al., 2021).

All participants in this study discussed their isolation at length. While specific questioning did inquire as to how the lockdown was impacting their mood, the majority of participants expressed worries about isolation and loneliness specifically, beyond that of introductory interview questions about mood. Participants expressed how the effects of isolation were a compounding factor for other mental health concerns; those with prior experience of depression and anxiety appeared to be particularly susceptible to the impacts of isolation as it triggered a recurrence of past negative thoughts that became worse over time. This is not surprising, as much work has been conducted into the ways in which mental health conditions can potentially reinforce each other under periods of heightened stress; specifically, Achterbergh et al. (2020) discussed how isolation significantly increased depressive symptoms in young adults and raised rates of depression meeting diagnosis thresholds. This further indicates the vulnerability of this population to the effects of lockdown, and the importance of monitoring the long-term potential impacts beyond the pandemic itself.

The overall theme of student mental health throughout this period could be described as “just managing”; students felt that they were not given the appropriate tools or support to properly flourish during their time at university and that they were being let down by the educational system as a whole. The overarching feeling was one of just getting through the academic year, as opposed to enjoying the university experience and everything it could potentially offer; a view highlighted in Turner et al. (2020) work studying the undergraduate experience in which they describe students needing to “learn loss” (pg 3346) to try and cope with the sense of a lost university experience.

Isolation then served to make this feeling worse, as students lost motivation to keep up with their workload, which fed back into the feeling of only just managing—it represents another kind of negative spiral that these students experienced during the pandemic. The future forecast for these students is one of potentially worse outcomes compared to their peers, and research has shown that students on a low-income background tend to graduate with fewer marks than their better-off peers (Carnevale & Smith, 2018), and the same is true for students with poor mental health (McKenzie & Schweitzer, 2001).

### Financial intersections

When openly invited to share any aspect of their lived experience during the pandemic, many participants raised the impact of money (this was unsolicited), and the perceived impact of income differences on student experiences. Participants talked of the inevitability of comparing themselves with their wealthier peers since these seemed so prominent in shaping how students lived and coped during the pandemic. The comparison was not about actual wealth differentials per se; participants also talked about concerns over whether they were good enough be at university, equating their sense of personal worth to their financial situation.

Negative self-worth has strong links with poor mental health. Campbell et al. (2022) systematic review examined studies that measured factors associated with student mental well-being and poor mental health in the UK student population, with 31 studies being included that were published within the last decade (2010–2020). Results indicated that poor self-worth was associated with poor mental health at university and was additionally found to be much more of a risk in higher education students and those who had prior experience with poor mental health than in a comparative group of young people not attending university. This highlights the way in which multiple vulnerabilities can interact to create a worsening of mental health for vulnerable students; coming from a low-income background can create a negative self-view, which adds to their past experience of poor mental health and ultimately places them at higher risk than other young people in the general population.

Concerns that the pandemic made their degree less valuable added to their existing anxieties about the high cost of attending university to begin with. This concern stemmed from the worry that employees would look down on their so-called “Covid cohort” as being the first group of students to graduate under such circumstances; that their degrees would be viewed less-favourably when compared to those not achieved under the pandemic—a worry shared by students beyond the UK, as statistics from the US (Klebs et al., 2021) cite that 56% of university students studying under COVID-19 felt anxiety over the worth of their degree. These worries, based on the materiality of low incomes, intersected with student mental health. Prior orientations to low mood or anxiety were stirred up by the often-overwhelming tensions and dilemmas around health vs wealth and the frustrations about the inadequacy of their degree experience.

This struggle to balance finances, and the mental health challenges that come about as a result, are further exacerbated by the changing political landscape within which students living in lower income brackets are expected to study and learn. As political choices are made to increase the cost of university education in the UK and prescribe limits of “low-worth degrees” (Hinds, 2019), students from lower-income backgrounds are forced now—more than ever—to examine the costs vs benefits of attending higher education. Participants also reported feeling that their degree was not worth the financial burden as it did not deliver the quality of education they felt they were paying for, even under pandemic conditions.

### Learning intersections

This study highlighted how the cumulative effects of lockdown and remote learning led to university students experiencing a powerful sense and fear of academic loss. This, in turn triggered reduced motivation to engage with new learning behaviours as they were often overwhelmed by new learning guidelines, and experienced self-doubt that led to disengagement with their studies.

According to a large-scale study of 1328 university students across 11 countries, including the UK, US, Canada, and Pakistan—where the study originated—on this student reaction to e-learning during the pandemic (Abbasi et al., 2020), higher education institutional pivots towards remote learning have been tumultuous and uncertain for students as worries during the pandemic over lack of routine, structure and support have been mete out in terms of how students have responded to the new educational environment. This supports initial concerns that extensive learning deficits would be evident as a result of the pandemic. The paper also drew focus to the way that students from disadvantaged social backgrounds were especially impacted, as remote learning was found to exacerbate educational inequalities between those from lower vs higher income backgrounds, which were already significant before the pandemic. This links with the present study's data as such social inequalities were observed to be a key factor in terms of the student academic experience, as many participants reported feeling particularly disadvantaged when compared to their better-off peers.

Motivation was another key factor that was frequently highlighted as problematic for the study participants as they had to balance their employment and academic work, with exhaustion often sapping the drive of participants who felt they did not have enough energy to maintain both aspects of their work life. This challenge of motivation extends to students with past experiences of poor mental health as mental health symptoms such as shame or lack of self-belief can have negative consequences for a person's sense of motivation (Kotera et al., 2019), but it is within the context of remote and/or hybrid learning that these consequences become more profound for students. A potential barrier against low motivation and mood is the positive presence of other people; through socializing, or simply by being close with, like-minded individuals a students can find renewed drive in the psychological “energy” of that shared physical space (Zhao et al., 2021). While virtual classrooms are trying to bridge this gap in socialization, participants in the present study discussed how they experienced a lack of belonging or togetherness with their course mates, and their university as a whole owing to the physical distance. They were not able to form that physical connection to the place or people.

Participants who find themselves in such vulnerable positions as being on a low income, or who are otherwise isolated, also experienced a powerful sense of having been forgotten about and neglected by university institutions. This placed great strain on motivation, as students expressed concern over the effectiveness of university provision during the lockdown period. Aguilera-Hermida (2020) reported findings echoing the present study, highlighting that US-based students felt abandoned and let down by university administrators in terms of the online learning environment being less engaging and lacking a defined source of support during the pandemic. These factors help in explaining why some students could not simply continue as normal. They experience a cognitive dissonance between their expectations of the university experience and the expectations placed upon them by the university and therefore are not properly supported to adapt to a fragile present and future; they feel a profound sense of abandonment and experience a great deal of self-criticism and self-doubt.

These harsh self-judgements led to students in the present study feeling that they were languishing, not flourishing; existing, as opposed to living. The current way that education is being delivered seems to be failing those students who need more support, and the support structure is not yet in place to help mitigate these impacts. According to Carl Rogers' Actualizing Theory (Rogers, 2008), all people have an innate need to grow and strive for more. This growth appears to have been restricted by the limitations of the new university environment students have been placed in as the remote environment is often not suitable for those who either face additional mental health challenges, or who are from a low-income background. In terms of the latter, disadvantaged students may not have the financial means to practically support their own learning—laptops and secure internet connections appear to be assumed by university policies as more courses become fully remote. Bonal and González (2020) highlighted the importance of acknowledging this potential lack of resources through their large-scale study of 35,419 families exploring the impact of remote learning on the learning gap of poor vs wealthy students. Overall, 25% of families in the lowest income quintile had access to only one digital device, compared to only 4% for families in the highest quintile. When considering the size of the surveyed households, 71% of the poorest families did not have access to one device per person. This highlights that significant inequalities in a student's ability to engage with distance learning are present in relation to income characteristics, which is concerning as universities are increasingly turning towards a hybrid or remote educational model.

Key questions raised as a result of this study include whether universities can/should be doing more to help bridge this income inequality, given that it directly impacts student engagement, learning and mental health. A dynamic system has emerged where anxiety, motivation, risk, and financial burdens intersect to create a perfect storm where great strain is placed on the individuals' mental health. Most institutions arguably do not know the extent that students experience these difficulties, and by drawing focus to these experiences, research can sensitize the institutions more to them to at least mitigate the impact.

### Limitations

Findings from this study should be considered in the light a number of limitations. Firstly, our sample self-defined as having experienced periods of low mood in the past, therefore we cannot claim our sample represents a clinical population. Our decision to use “low mood” and “poor mental health” was based on likely meaningfulness to potential participants which was appropriate for this study but is nonetheless a limitation in its design. Interviews often surfaced quite complex mental health experiences among participants, with comorbidities also being discussed—our focus was not on the presence of these additional experiences, meaning that some avenues of exploration were necessarily cut short.

Other limitations then are that the experiences reported were infused with the broader national and global reflections on the impact of the pandemic, as well as national debates about how the higher education sector should respond and if they were behaving well enough. While such contexts were important in terms of understanding and appreciating participants' experiences, they also shaped the way in which they were analyzed and investigated. Context is vital for qualitative study; however, it is also important that it not overtake the story being told (Loudoun & Townsend, 2024). While efforts were made to avoid this, the overarching impact of the pandemic context could have impacted the richness of analysis.

### Recommendations for practice

Universities have a responsibility to mitigate against inequalities and make reasonable adjustments to their learning methods to support those students whose needs are different to the majority (Pactwa et al., 2024). The pandemic shone a revealing light on the pitfalls of current provision (Rowland, 2022) and the negative experiences of vulnerable students during challenging periods (Schiff et al., 2024).

Firstly, it is recommended that universities should make plans for future pandemics or other lockdown scenarios. There remains a very real risk that further pandemics may occur as COVID-19 variants continue to be monitored. This means that the potential for future lockdowns must not be ignored, and universities should learn from the studies such as this one to improve outcomes for vulnerable students. One way to achieve this could be to adopt greater transparency in university communication—giving students more clear information on what decisions are being made and why.

Another vital area to improve is the sense of online community that students felt was largely absent during the lockdown period. As many universities continue to use hybrid or remote learning models, many students remain living away from campus and away from in-person learning and socializing opportunities that can leave them feeling disillusioned and distant from their institution. It is undoubtedly difficult to foster a similar sense of community online to that which forms in-person; however, the shift to virtual environment necessitates improvements in this area. Improving the ease at which remote students can freely communicate with both their peers and staff could help mitigate this lack of engagement—using instant chat rooms linked with peer groups which could use remote verbal and written communication to help bring students together.

In terms of students who come to university with past experiences of poor mental health, it is recommended that these students be given priority invitations to engage with mental health support services on campus, as opposed to having to take that first step themselves. It can be difficult for a person facing mental health challenges to reach out and ask for help—as was the case in this study—and by removing that initial barrier, there is potential for services to reach those students who need it the most. Such services can include counselling, mental health mentors, or academic/pastoral support.

This study set out to explore the lived experiences and inequalities faced by vulnerable UK university students during the pandemic, seeking to identify how vulnerabilities interacted to impact their thoughts, feelings, and learning experience. Key findings highlight a cohort of students who feel isolated, lonely, and forgotten by institutions, left to languish where they should be flourishing; whose financial circumstances make those mental health experiences even more challenging; and whose learning experience has been one of disruption, upset, and unmet expectation.

It is vital that other vulnerable students be supported as young adults emerge into a world beyond university that they feel unprepared for, and as such institutions must work with students to offer a more visible, student-focused support structure in order to address both the mental health and financial burden that students find themselves carrying.

## Data Availability

Data are available upon request. It was not deposited in a repository to assist in reassuring participant data security and ownership.

## References

[cit0001] Abbasi, M. S., Ahmed, N., Sajjad, B., Alshahrani, A., Saeed, S., Sarfaraz, S., & Abduljabbar, T. (2020). E-Learning perception and satisfaction among health sciences students amid the COVID-19 pandemic. *Work*, *67*(3), 549–556. 33185620 10.3233/WOR-203308

[cit0002] Achterbergh, L., Pitman, A., Birken, M., Pearce, E., Sno, H., & Johnson, S. (2020). The experience of loneliness among young people with depression: A qualitative meta-synthesis of the literature. *BMC Psychiatry*, *20*(1), 415. 10.1186/s12888-020-02818-3 32831064 PMC7444250

[cit0003] Aguilera-Hermida, A. P. (2020). College students' use and acceptance of emergency online learning due to COVID-19. *International Journal of Educational Research Open*, *1*, 100011. 10.1016/j.ijedro.2020.100011 35059662 PMC7480788

[cit0004] Ali, I., & Khattak, I. (2023). Intersectionality in mental health: exploring the complexities of identity and well-being. *Journal Of Psychology, Health And Social Challenges*, *1*(01), 43–54.

[cit0005] Bonal, X., & González, S. (2020). The impact of lockdown on the learning gap: family and school divisions in times of crisis. *International Review of Education*, *66*(5-6), 635–655. 10.1007/s11159-020-09860-z 32952208 PMC7490481

[cit0006] Braveman, P., & Gruskin, S. (2003). Defining equity in health. *Journal of Epidemiology & Community Health*, *57*(4), 254–258. 10.1136/jech.57.4.254 12646539 PMC1732430

[cit0007] Campbell, F., Blank, L., Cantrell, A., Baxter, S., Blackmore, C., Dixon, J., & Goyder, E. (2022). Factors that influence mental health of university and college students in the UK: A systematic review. *BMC Public Health*, *22*(1), 1–22. 10.1186/s12889-022-13943-x 36123714 PMC9484851

[cit0008] Carnevale, A. P., & Smith, N. (2018). Balancing work and learning: Implications for low-income students.

[cit0009] Claes, N., Smeding, A., & Carré, A. (2021). Mental health inequalities during COVID-19 outbreak: The role of financial insecurity and attentional control. *Psychologica Belgica*, *61*(1), 327. 10.5334/pb.1064 34824863 PMC8588930

[cit0010] Coombs, N. C., Meriwether, W. E., Caringi, J., & Newcomer, S. R. (2021). Barriers to healthcare access among US adults with mental health challenges: A population-based study. *SSM-population health*, *15*, 100847. 10.1016/j.ssmph.2021.100847 34179332 PMC8214217

[cit0011] Coyne, C., Ballard, J. D., & Blader, I. J. (2020). Recommendations for future university pandemic responses: What the first COVID-19 shutdown taught us. *PLoS Biology*, *18*(8), e3000889. 10.1371/journal.pbio.3000889 32853196 PMC7480850

[cit0012] Dougall, I., Vasiljevic, M., Kutlaca, M., & Weick, M. (2023). Socioeconomic inequalities in mental health and wellbeing among UK students during the COVID-19 pandemic: Clarifying underlying mechanisms. *PLoS One*, *18*(11), e0292842. 10.1371/journal.pone.0292842 37910542 PMC10619810

[cit0013] Eatough, V., & Smith, J. A. (2017). Interpretative phenomenological analysis. *The Sage handbook of qualitative research in psychology*. 193–209. 10.4135/9781526405555.n12

[cit0014] Engward, H., Goldspink, S., Iancu, M., Kersey, T., & Wood, A. (2022). Togetherness in separation: Practical considerations for doing remote qualitative interviews ethically. *International Journal of Qualitative Methods*, *21*. 16094069211073212. 10.1177/16094069211073212

[cit0015] Espinoza, O. (2007). Solving the equity–equality conceptual dilemma: A new model for analysis of the educational process. *Educational Research*, *49*(4), 343–363. 10.1080/00131880701717198

[cit0016] Farquharson, C., McNally, S., & Tahir, I. (2024). Education inequalities. *Oxford Open Economics*, *3*(Supplement_1), i760–i820. 10.1093/ooec/odad029

[cit0017] Finlay, L. (2011). *Phenomenology for therapists: Researching the lived world*. John Wiley & Sons.

[cit0018] Gillborn, D., Warmington, P., & Demack, S. (2012). QuantCrit: Education, policy, ‘Big data’ and principles for a critical race theory of statistics. *Race Ethnicity and Education*, *21*(2), 158–179. 10.1080/13613324.2017.1377417

[cit0019] Government Equalities Office. (2015). *Equality Act 2010: Guidance*. Accessed from https://www.gov.uk/guidance/equality-act-2010-guidance

[cit0020] Guest, G., MacQueen, K. M. (Eds.). (2008). *Handbook for team-based qualitative research*. Rowman Altamira.

[cit0021] Gustavson, K., von Soest, T., Karevold, E., & Røysamb, E. (2012). Attrition and generalizability in longitudinal studies: Findings from a 15-year population-based study and a Monte Carlo simulation study. *BMC Public Health*, *12*(1), 1–11. 10.1186/1471-2458-12-918 23107281 PMC3503744

[cit0022] Hinds, D. (2019). Education Secretary calls for an end to low-value degrees. https://www.gov.uk/government/news/education-secretary-calls-for-an-end-to-low-value-degrees

[cit0023] Horner, C. R. (2023). Understanding mental health, educational risk and resilience for vulnerable first-year students during the Covid-19 pandemic: A UK-based, longitudinal qualitative study. PhD thesis, University of Leeds.

[cit0024] Huckins, J. F., DaSilva, A. W., Wang, W., Hedlund, E., Rogers, C., Nepal, S. K., Campbell, A. T., Wu, J., Obuchi, M., Murphy, E. I., Meyer, M. L., Wagner, D. D., & Holtzheimer, P. E. (2020). Mental health and behavior of college students during the early phases of the COVID-19 pandemic: Longitudinal smartphone and ecological momentary assessment study. *Journal of Medical Internet Research*, *22*(6), e20185. 10.2196/20185 32519963 PMC7301687

[cit0025] Klebs, S., Fishman, R., Nguyen, S., & Hiler, T. (2021). One year later: COVID-19s impact on current and future college students. Accessed from https://www.luminafoundation.org/wp-content/uploads/2021/08/one-year-later.pdf

[cit0026] Kotera, Y., Conway, E., & Van Gordon, W. (2019). Mental health of UK university business students: Relationship with shame, motivation and self-compassion. *Journal of Education for Business*, *94*(1), 11–20. 10.1080/08832323.2018.1496898

[cit0027] Leal Filho, W., Wall, T., Rayman-Bacchus, L., Mifsud, M., Pritchard, D. J., Lovren, V. O., Balogun, A. L., Farinha, C., & Petrovic, D. S. (2021). Impacts of COVID-19 and social isolation on academic staff and students at universities: A cross-sectional study. *BMC Public Health*, *21*(1), 1213. 10.1186/s12889-021-11040-z 34167494 PMC8223197

[cit0028] Liu, F., Dai, L., Cai, Y., Chen, X., Li, J., & Shi, L. (2023). Psychological state and its correlates of local college students in wuhan during COVID-19 pandemic. *Psychology in the Schools*, *60*(5), 1477–1487. 10.1002/pits.22699 PMC9088483 35572182

[cit0029] Loudoun, R., & Townsend, K. (2024). What precisely do you mean? Interpreting qualitative data, *How to Keep Your Research Project on Track*. 111–118. Edward Elgar Publishing. 10.4337/9781035332724.00027

[cit0030] McCloud, T. (2022). The mental health of higher education students and the role of finances and debt (Doctoral dissertation, UCL (University College London)).

[cit0031] McKenzie, K., & Schweitzer, R. (2001). Who succeeds at university? Factors predicting academic performance in first year Australian university students. *Higher Education Research & Development*, *20*(1), 21–33. 10.1080/07924360120043621

[cit0032] Miller, R. M., Chan, C. D., & Farmer, L. B. (2018). Interpretative phenomenological analysis: A contemporary qualitative approach. *Counselor Education and Supervision*, *57*(4), 240–254. 10.1002/ceas.12114

[cit0033] O'Connor, R. C., Perry, V. H., Tracey, I., Wessely, S., Arseneault, L., Ford, T., Hotopf, M., Worthman, C. M., Ballard, C., Christensen, H., Silver, R. C., John, A., Kabir, T., King, K., Simpson, A., Madan, I., Cowan, K., Bullmore, E., & Holmes, E. A. (2020). Multidisciplinary research priorities for the COVID-19 pandemic: A call for action for mental health science. *The Lancet Psychiatry*, *7*, e44–e45. 10.1016/S2215-0366(20)30247-9 32563319 PMC7302786

[cit0034] Pactwa, K., Woźniak, J., Jach, K., & Brdulak, A. (2024). Including the social responsibility of universities and sustainable development goals in the strategic plans of universities in Europe. *Sustainable Development*, *32*(5), 4593–4605. 10.1002/sd.2924

[cit0035] Phillippi, J., & Lauderdale, J. (2018). A guide to field notes for qualitative research: Context and conversation. *Qualitative Health Research*, *28*(3), 381–388. 10.1177/1049732317697102 29298584

[cit0036] Richards, K. A. R., & Hemphill, M. A. (2018). A practical guide to collaborative qualitative data analysis. *Journal of Teaching in Physical education*, *37*(2), 225–231. 10.1123/jtpe.2017-0084

[cit0037] Richardson, T., Sood, M., Large, J., & McCloud, T. (2024). The impact of the 2012 student fees increase on the mental health of British graduates: A cohort study. *Journal of Public Mental Health*, *23*(4), 330–338. 10.1108/JPMH-08-2024-0105

[cit0038] Roberts, J. K., Pavlakis, A. E., & Richards, M. P. (2021). It's more complicated than it seems: Virtual qualitative research in the COVID-19 era. *International Journal of Qualitative Methods*, *20*, 16094069211002959. 10.1177/16094069211002959

[cit0039] Robertson, A., Mulcahy, E., & Baars, S. (2022). What works to tackle mental health inequalities in higher education. Transforming Access and Student Outcomes in Higher Education.

[cit0040] Rogers, C. R. (2008). The actualizing tendency in relation to'motives' and to consciousness, *In Nebraska Symposium on Motivation* (Vol. *1963*). Pccs Books Reprinted from the aforementioned conference.

[cit0041] Rowland, A. (2022). Responses to the pandemic in higher education: An EA report (2022). The English Association. https://englishassociation.ac.uk/responses-to-the-pandemic-in-higher-education-an-ea-report-2022/

[cit0042] Salimi, N., Gere, B., Talley, W., & Irioogbe, B. (2023). College students mental health challenges: Concerns and considerations in the COVID-19 pandemic. *Journal of College Student Psychotherapy*, *37*(1), 39–51. 10.1080/87568225.2021.1890298

[cit0043] Schiff, M., Pat-Horenczyk, R., & Benbenishty, R. (2024). University students coping with COVID-19 challenges: Do they need help? *Journal of American College Health*, *72*(2), 578–586. 10.1080/07448481.2022.2048838 35271417

[cit0044] Smith, J. A., Flowers, P., & Larkin, M. (2014). *Analysis: Theory, method and research*.

[cit0045] Smith, J. A., Flowers, P., & Larkin, M. (2021). *Interpretative phenomenological analysis: Theory, method and research*. SAGE.

[cit0046] Son, C., Hegde, S., Smith, A., Wang, X., & Sasangohar, F. (2020). Effects of COVID-19 on college students' mental health in the United States: Interview survey study. *Journal of Medical Internet Research*, *22*(9), e21279. 10.2196/21279 32805704 PMC7473764

[cit0047] Subu, M. A., Wati, D. F., Netrida, N., Priscilla, V., Dias, J. M., Abraham, M. S., Al-Yateem, N., & Slewa-Younan, S. Types of stigma experienced by patients with mental illness and mental health nurses in Indonesia: A qualitative content analysis. *International Journal of Mental Health Systems*, *15*, 1–12. 34663399 10.1186/s13033-021-00502-x PMC8524985

[cit0048] Turner, K. L., Hughes, M., & Presland, K. (2020). Learning loss, a potential challenge for transition to undergraduate study following COVID19 school disruption. *Journal of Chemical Education*, *97*(9), 3346–3352. 10.1021/acs.jchemed.0c00705

[cit0049] UNESCO. (2020). Five years of impact: UNESCO's Global Education Coalition highlights key milestones. Accessed from https://www.unesco.org/en/articles/five-years-impact-unescos-global-education-coalition-highlights-key-milestones

[cit0050] UNESCO. (2025). UN secretary-general warns of education catastrophe, pointing to UNESCO estimate of 24 million learners at risk of dropping out. Accessed from https://www.UNESCO.org/en/articles/un-secretary-general-warns-education-catastrophe-pointing-UNESCO-estimate-24-million-learners-risk-0

[cit0051] Zhao, L., Cao, C., Li, Y., & Li, Y. (2021). Determinants of the digital outcome divide in E-learning between rural and urban students: Empirical evidence from the COVID-19 pandemic based on capital theory. *Computers in Human Behavior*, *130*, 107177. 10.1016/j.chb.2021.107177 36568533 PMC9758626

